# A handheld device for measuring the diameter at breast height of individual trees using laser ranging and deep-learning based image recognition

**DOI:** 10.1186/s13007-021-00748-z

**Published:** 2021-06-25

**Authors:** Chuangye Song, Bin Yang, Lin Zhang, Dongxiu Wu

**Affiliations:** 1 State Key Laboratory of Vegetation and Environmental Change, Institute of Botany, Chinese Academy of Sciences, Beijing, 100093 China; 2 Yusense Information Technology and Equipment Inc., Qingdao, 266111 China

**Keywords:** Forest inventory, Tree measurement, Digital camera, Convolutional neural networks, Spatial attention module

## Abstract

**Background:**

Accurate and efficient measurement of the diameter at breast height (DBH) of individual trees is essential for forest inventories, ecological management, and carbon budget estimation. However, traditional diameter tapes are still the most widely used dendrometers in forest surveys, which makes DBH measurement time-consuming and labor-intensive. Automatic and easy-to-use devices for measuring DBH are highly anticipated in forest surveys. In this study, we present a handheld device for measuring the DBH of individual trees that uses digital cameras and laser ranging, allowing for an instant, automated, and contactless measurement of DBH.

**Results:**

The base hardware of this device is a digital camera and a laser rangefinder, which are used to take a picture of the targeted tree trunk and record the horizontal distance between the digital camera and the targeted tree, respectively. The core software is composed of lightweight convolutional neural networks (CNNs), which includes an attention-focused mechanism for detecting the tree trunk to log the number of pixels between the edges. We also calibrated the digital camera to correct the distortion introduced by the lens system, and obtained the normalized focal length. Parameters including the horizontal distance between the digital camera and the targeted tree, number of pixels between the edges of the tree trunk, and normalized focal length were used to calculate the DBH based on the principles of geometrical optics. The measured diameter values, and the longitudes and latitudes of the measurement sites, were recorded in a text file, which is convenient to export to external flash disks. The field measurement accuracy test showed that the BIAS of the newly developed device was − 1.78 mm, and no significant differences were found between the measured diameter values and the true values (measured by the conventional tape). Furthermore, compared with most other image-based instruments, our device showed higher measurement accuracy.

**Conclusions:**

The newly developed handheld device realized efficient, accurate, instant, and non-contact measurements of DBH, and the CNNs were proven to be successful in the detection of the tree trunk in our research. We believe that the newly developed device can fulfill the precision requirement in forest surveys, and that the application of this device can improve the efficiency of DBH measurements in forest surveys.

## Background

Forest inventory is an important approach for determining the quantity, quality, and distribution of forest resources [1]. In a forest survey, the diameter at breast height (DBH) of individual trees is one of the most important indicators of tree attributes [2]. Accurate measurement of DBH is essential for forest resource inventory and management, tree growth, and carbon cycle modeling [1, 3]. Currently, acquiring the DBH of individual trees using traditional tapes is a time-consuming and labor-intensive endeavor. Devices which can obtain DBH in a rapid and accurate manner are highly anticipated [4].

Methods for measuring DBH can be divided into two categories: contact and non-contact. Contact dendrometers need to physically touch the tree trunk. Conventional calipers and diameter tapes are the most widely used contact dendrometers in forest surveys. Usually, two people are required to perform the DBH measurement (one for measuring, the other for recording). The limitations of contact dendrometers are their low efficiency and high labor cost. Non-contact dendrometers, such as optical calipers [5], rangefinder dendrometers [6], and optical forks [7], have been designed based on the principle of optical measurement. They do not need to touch the tree trunk; instead, perspective geometry utilizes various angles and distances to calculate the trunk diameter [8]. Photographs taken using a conventional film camera have also been used to perform non-contact DBH measurements. However, additional tools, such as reference stick with a known length or control points, must be placed near the tree trunk as a reference scale in order to determine how distances on the image relate to those in the real world, before the DBH of the tree trunk can be calculated [9, 10]. Moreover, lens distortions and film non-flatness from cameras can decrease the accuracy of the DBH measurements [11].

With the development of digital imaging technology, digital cameras have been used to measure the DBH of individual trees. These methods commonly require auxiliary tools such as reference sticks and calibration poles, and the contours of tree trunks are usually extracted manually, or through color-based approaches. For example, Clark [4] developed an instrument to measure DBH that incorporated a digital camera, a 3-axis magnetometer, and a laser rangefinder. The photo of the tree, range, and orientation data were fed into the “Tree Measurement System” processing program to calculate parameters such as DBH, height, and stem volume. However, manual input was still required to extract the contour of the tree trunk. Juujärvi et al. and Varjo et al. [12, 13] developed an image-based tree measurement system, which consisted of a digital camera, a laser rangefinder, and a calibration stick. Color and stem form models were combined to create a histogram separation model to locate the trunk curves and automatically extract the trunk frame. Camera geometry parameters and viewing geometry must first be determined before the color image information can be transformed into a three-dimensional trunk model of the tree and yield the measurements of tree height and DBH. Brownlie et al. [11] designed a photogrammetric image-based dendrometry system called “TreeD” for measuring the features of individual standing trees. Additional tools such as a transponder and height pole are needed for the “TreeD” system. Field parameters, including the horizontal distance from the camera to the tree and the height of the transponder above the ground, must be measured in the field. These parameters are used to register the tree images in a three-dimensional space using complex triangular-geometry calculations and coordinate transformations. Parameters such as DBH, height, and crown size, can then be measured in the “TreeD” system using stereogram-displaying software. Gazda and Kedra [14] developed a tree architecture description method using an image photogrammetric method, which includes image transformation (turning a non-metric into a metric image), calibration with a reference object, and vectorization. In recent years, smartphone-based passive monocular vision measurement methods have also been used to measure the DBH. For example, Wu et al. [3] proposed a method for measuring the DBH of multiple trees based on a single image taken by a smartphone camera, using machine vision and close-range photogrammetry technology. According to Wu et al., a visual segmentation approach based on an improved frequency-tuned saliency algorithm was used to extract the trunk contour using the color features. An adaptive feature coordinate system and the color information of the tree trunk were used to measure DBH.

Several studies have attempted to utilize multiple images taken from different directions to generate point-cloud data to measure the DBH of individual trees at the plot level. For example, Liang et al. [15] collected several photos taken at different positions around a forest plot using an uncalibrated digital camera. These photographs were used to generate point-cloud data by utilizing the automated image matching process of the Agisoft PhotoScan professional commercial software. The point-cloud data in the camera space was then transformed to obtain 3-Dimensional (3D) point-cloud data in the real-word space, which was then used to measure the DBH of each individual tree in the plot. Mulverhill et al. [16] also used the Agisoft PhotoScan software to construct accurate photogrammetric point-cloud data, and derived DBH, height, taper, and volume of trees in a plot. Forsman et al. [17] utilized a prototype multi-camera rig to record images from the center of field plots in multiple directions. Images were then used to generate point-cloud data to estimate tree attributes. Fan et al. [18] used a smartphone with a Google Tango sensor (the smartphone contained a combination of an RGB (red, green, blue) camera, a time-of-flight camera, and a motion-tracking camera called a vision sensor) to record images of trees, and they designed an algorithm to estimate the DBH and the location of the trees in the plot, using the point-cloud data generated from the time-of-flight camera and camera pose. The advantages of image-based point-cloud data include the low price of the equipment and the simplicity of the field measurements, and the disadvantages include the difficulties of mapping small trees and trees that are occluded by the complex forest stands, and the time required for data processing [15].

In past few years, with the development of light detection and ranging (LiDAR) technology, more and more research has utilized ground-based or unmanned LiDAR scanning to obtain 3D point-cloud data of trees, and to derive height and DBH measurements [19–23]. The advantage of LiDAR technology is that it can describe the 3D structure of trees and obtain multiple tree parameters (such as height, DBH, and crown size) at the plot level. However, LiDAR equipment is expensive, its operation in the field is complicated, and data processing is very complex and specialized. At present, it is still difficult to utilize LiDAR technology widely in forestry surveys [21].

Based on the above discussion, we can see that reference sticks, calibration poles, and auxiliary indicators such as angles and distances are needed in the early image-based measurements of DBH. Manual processing is required to extract the trunk contour. This leads to a low degree of automation in measuring DBH. Furthermore, the calculation stage for many prior instruments needed to be conducted on a computer [4, 11–13], which led to low working efficiency in field forest surveys. Presently, smartphone-based machine vision and close-range photogrammetry technology have improved the degree of automation in image-based DBH measurements. Reference sticks and calibration poles are rarely used in field measurements. However, conversions between different coordinate systems (e.g., image plane coordinate systems, image space coordinate systems, photogrammetric coordinate systems, and object space coordinate systems) are quite complicated. Furthermore, the accuracy of the three-dimensional coordinates derived from two-dimensional image coordinates cannot be guaranteed [24], which decreases the accuracy of DBH measurements. It is worth mentioning that trunk contour extraction is a vital step for measuring DBH. However, in current research, most algorithms (such as histogram comparisons) of trunk contour extraction is based on the color information of the trunk, which is prone to error in the identification of tree trunks. In addition, instruments developed in previous works were mostly a loose collection of different hardware (e.g., a digital camera, a laser rangefinder, a transponder, a tripod, and a calibration pole), and no highly integrated and handheld device has been developed for easy and convenient DBH measurements. A compact design and user-friendly device could bring image-based DBH measurements to a wider range of users. Therefore, in this research, we attempted to develop a handheld, highly integrated DBH measurement device based on image recognition and laser ranging. We employed convolutional neural networks (CNNs) to identify the tree trunks using color and texture information. The newly developed device can record the longitudes and latitudes of the measurement sites in a text file format together with the measured DBH values and store this along with the tree images in the memory card. We believe that our device can improve the accuracy and efficiency of DBH measurements in forest resource surveys.

## Materials and methods

### General introduction

The proposed device uses laser ranging and image recognition, and has been developed to perform non-contact DBH measurements. The measured DBH values and the latitudes and longitudes of measurement sites were recorded and written into a text file, which can be easily transferred to an external flash disk.

**Fig. 1:**
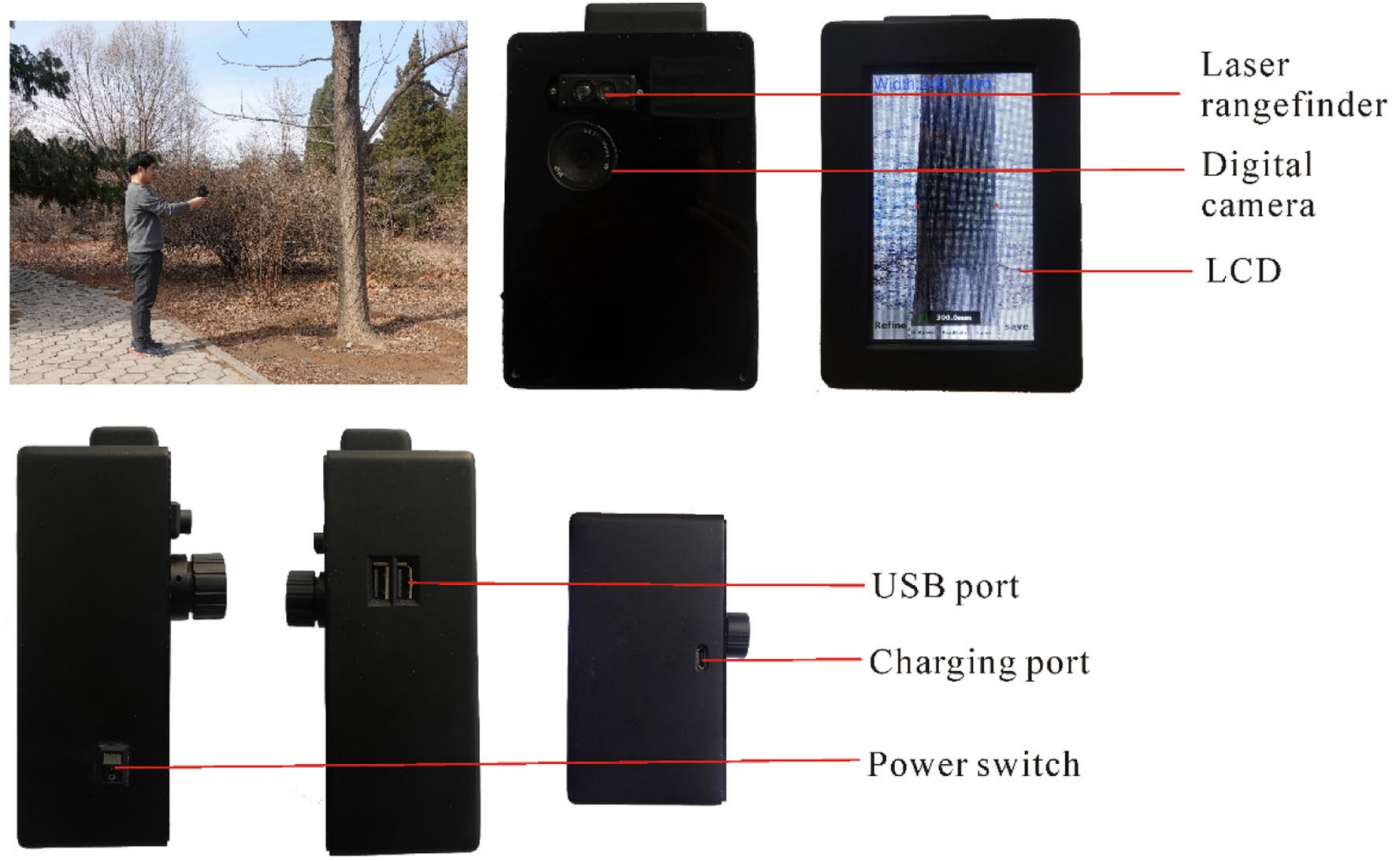
Structure of the device. *LCD* liquid crystal display, *USB* universal serial bus

The core software used is the object detection algorithm, which utilizes CNNs to precisely detect tree trunks. The core hardware includes a digital camera, a laser rangefinder, an embedded development board, a global positioning system (GPS), battery, liquid crystal display (LCD), and a memory module (Fig. 1). The size of the device is 10.5 cm × 5.5 cm × 14.5 cm (length × width × height), and the weight is 600 g, which is light enough to be carried by a single person operating in the field without the support of a tripod. This device can work continuously for about 12 h in an environment with a temperature range of 0–40 °C, which meets the requirements of field forest surveys.

**Fig. 2:**
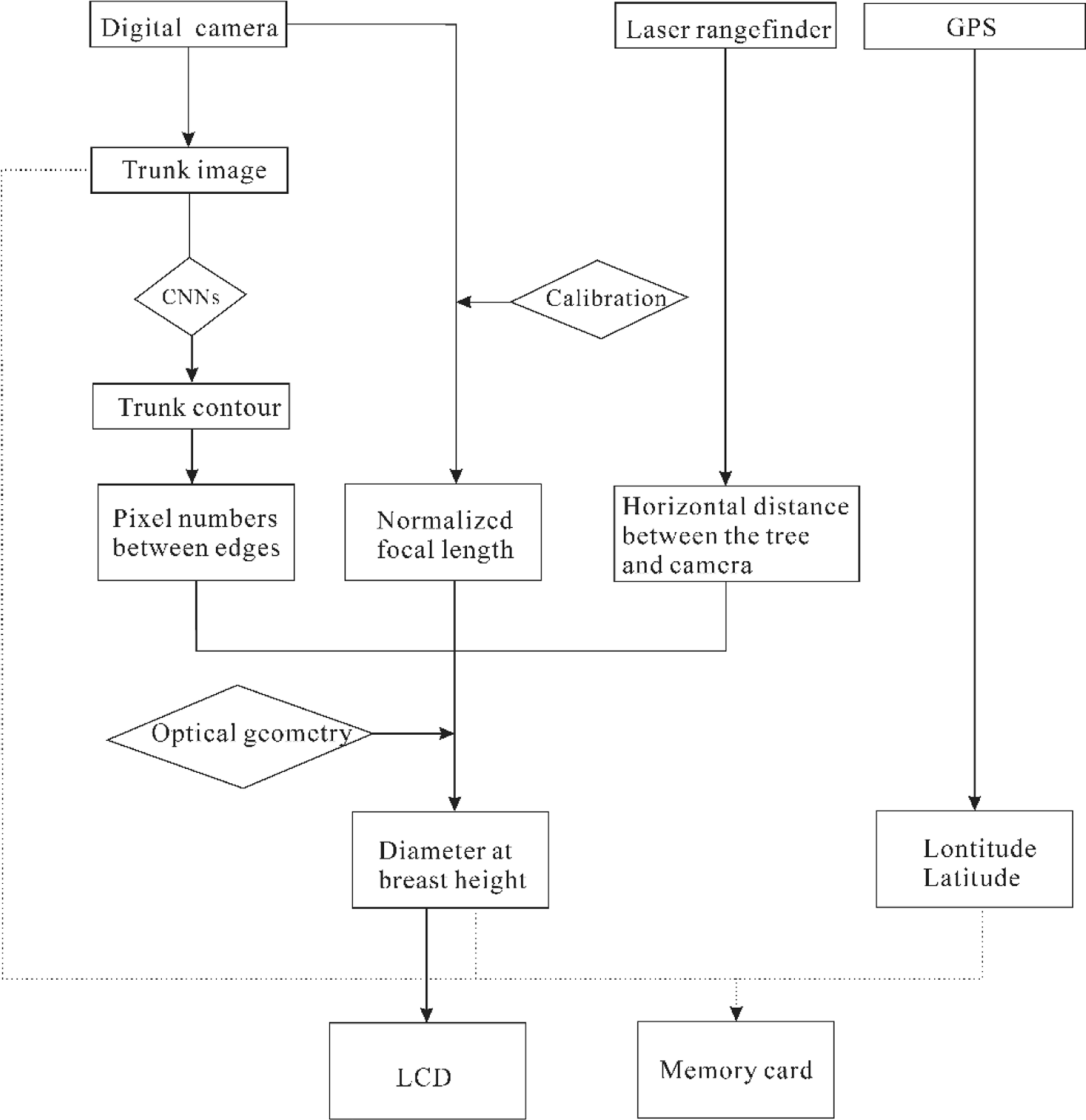
Workflow of the presented device. *CNNs* convolutional neural networks, *LCD* liquid crystal display, *GPS* global positioning system

When the device is powered on, the microprocessor continuously reads the low-resolution video through the interface of the digital camera, and a real-time video is displayed on the LCD. When the operator issues the “Take photo” command to the digital camera by pressing a virtual button on the LCD, the digital camera captures a high-resolution photo of the targeted tree trunk. The photo is then processed by the microprocessor to identify and extract the trunk using CNNs algorithm. Then, the number of pixels between the edges of the extracted trunk contour is recorded. Meanwhile, the laser rangefinder measures the horizontal distance between the digital camera and the targeted tree trunk. This information is sent to the microprocessor to calculate the DBH based on the theory of geometrical optics. The DBH value is then displayed on the LCD and written into a text file together with the recorded latitude and longitude. The workflow of the device is shown in Fig. 2.

### Theoretical basis

**Fig. 3:**
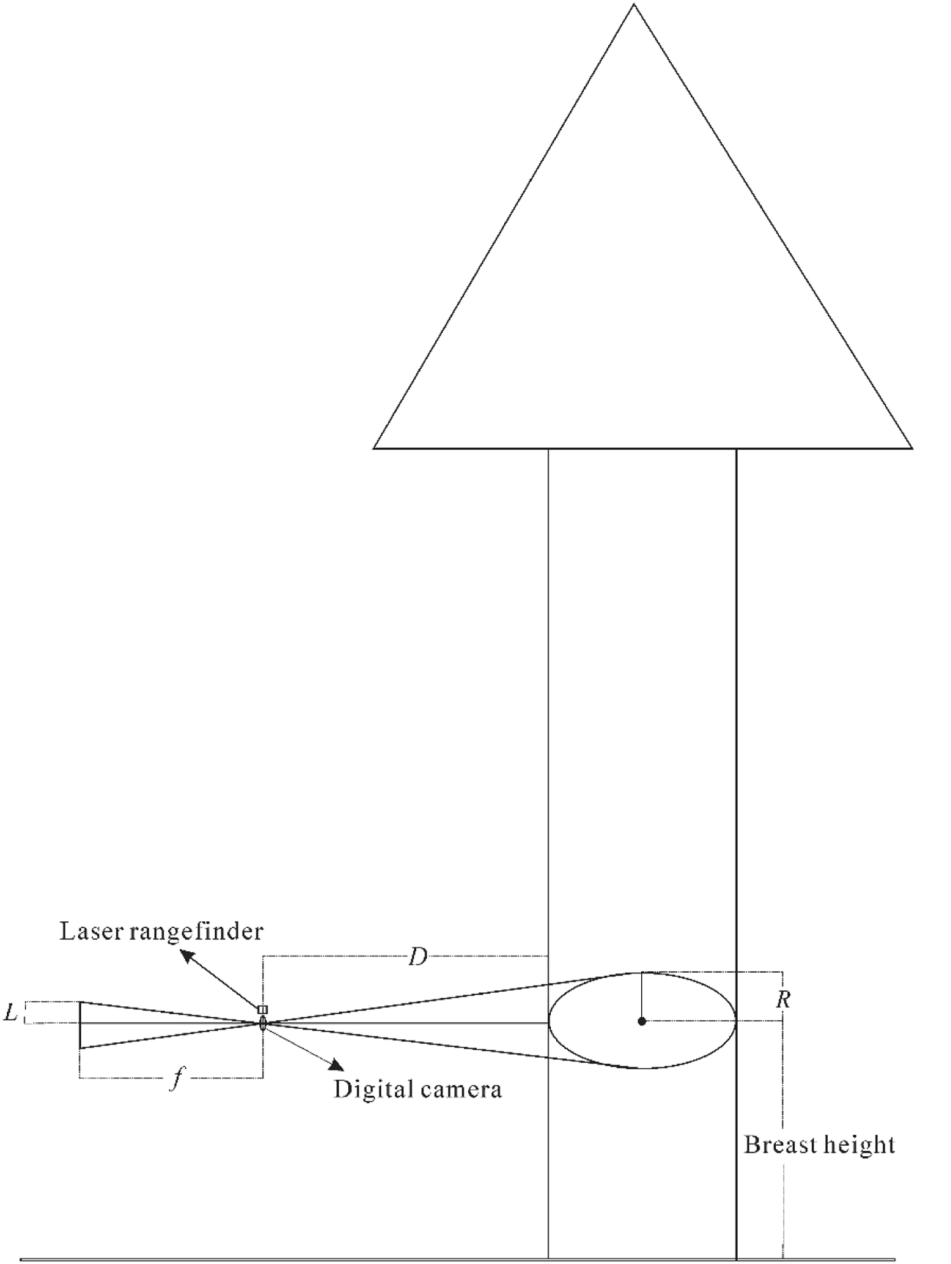
Working principle of the device

The theoretical basis of the proposed device is shown in Fig. 3. The DBH is measured based on the horizontal distance from the device to the targeted tree trunk, intrinsic camera parameters (focal length, pixel size), and number of pixels between the edges of the trunk at breast height. In Fig. 3, $L$ is the projection of the semidiameter of the trunk on the charge-coupled device (CCD) plate of the digital camera, ${ }f{ }$ is the focal length, $D$ is the horizontal distance from the camera to the targeted tree trunk, and *R* is the semidiameter of the tree trunk.

In the theory of geometrical optics, the relationships between $L$, ${ }f,$ and $D$ are expressed by Eq. (1). Based on the imaging principle of a digital camera, $L$ can be calculated using Eq. (2).

1 $$\frac{L}{f} = \frac{R}{R + D},$$

2 $$L = \frac{1}{2}\left( {N \times \mu } \right).$$

In Eq. (2), $N$ is the number of pixels between the edges of the tree trunk at breast height. $\mu$ is the pixel size. We can use Eq. (3) to calculate $R$ based on the combination of Eqs. (1) and (2).

3 $$R = \frac{DN\mu }{{2f - N\mu }}.$$

$f_{x}$ is the normalized focal length of the abscissa axis, which is calculated using Eq. (4). Based on Eqs. (3) and (4), we can use Eq. (5) to calculate $R$.

4 $$f_{x} = f/\mu ,$$

5 $$R = \frac{DN}{{2\mathop f\nolimits_{x} - N}},$$

${\text{f}}_{x}$ is one of the intrinsic camera parameters. Although the manufacturer has provided intrinsic parameters such as pixel size and focal length, we need to calibrate the camera to determine the precise intrinsic camera parameters. In this research, we used the method described by Zhang [25] to calibrate the camera and obtain the normalized focal length ($f_{x}$). $N$ is the number of pixels between the edges of the extracted tree trunk at breast height.

### Detection of the tree trunk

Detecting the tree trunk is a demanding task because of the variations in texture and color richness of the tree trunk, occlusions of forest scene objects, complex backgrounds, and diverse lighting conditions. The emergence of CNNs provides a good solution for object detection [26]. It can automatically acquire features from the training data that represent the nature of the target. Compared with manually selected features, deep features selected by CNNs have a robust ability to describe the characteristics of targeted objects [27]. Several researchers have utilized CNNs to detect objects in image interpretation [28–32]. In the present study, we adopted a lightweight algorithm based on CNNs that includes an attention-focused mechanism for detecting the tree trunk.

#### Dataset construction

CNNs are data-driven deep-learning algorithms that require sample data to train the model for object detection. We collected 200 pictures of trees, including those of *Cerasus serrulata*, *Amygdalus persica*, *Pinus tabuliformis*, *Ailanthus altissima*, and *Fraxinus chinensis*. We extracted sub-images of the tree trunks from these pictures manually, and used half of them as the training data, while the remaining images were used as the test data.

#### Construction of the CNNs

A large receptive field is the key to the effective extraction of semantic edges, and the size of the receptive field increases with increasing convolutional layers. The size of the receptive field is calculated using Eq. (6):

6 $$l_{k} = l_{k - 1} + \left[ {\left( {f_{k} - 1} \right) \times \mathop \prod \limits_{i = 1}^{k - 1} s_{i} } \right].$$

In Eq. (6), $l_{k - 1}$ is the size of the receptive field for the $k - 1$ convolutional layer, $f_{k}$ is the kernel or pool size of the $k$ layer, and $S_{i}$ is the stride of the convolution or pooling layer. The increase in the receptive field size can be achieved by either increasing the size of the kernel or the stride. However, increasing the size of the convolution kernel increases the computation load exponentially. It is difficult for an embedded device to accomplish this computing process. Therefore, we chose to increase the size of the receptive field by increasing the stride size of the convolution. Increasing the stride size can also reduce the size of the feature map and effectively decrease the amount of computation.

Pool down-sampling and convolution down-sampling are two approaches that are commonly used to increase the stride size. As pool down-sampling is more conducive to model convergence, we chose pool down-sampling in the CNNs to increase the stride size. Although we reduced the computation load by pool down-sampling, the computation overhead and memory overhead were still very large for an embedded device. Therefore, we took measures to further compress the CNNs. In our research, we utilized the same approach adopted by MobileNet [33] to compress the model, using separable convolution instead of standard convolution filters to process the information. Separable convolution was composed of depth wise convolutions and 1 × 1 convolutions.

The number of parameters (Prams) and the cost of the standard convolution (Cost) can be obtained by Eqs. (7) and (8) respectively.

7 $${\text{Prams}} = D_{k} \times D_{k} \times M \times N,$$

8 $${\text{Cost}} = D_{k} \times D_{k} \times M \times N \times D_{f} \times D_{f} .$$

In Eqs. (7) and (8), $D_{k}$ is the size of the convolution kernel, $M$ is the number of input channels, $N$ is the number of output channels, and $D_{f}$ is the size of the feature map.

The number of parameters and the cost of the separable convolution can be obtained separately using Eqs. (9) and (10).

9 $${\text{Prams}} = D_{k} \times D_{k} \times M + 1 \times 1 \times M \times N,$$

10 $${\text{Cost}} = D_{k} \times D_{k} \times M \times D_{f} \times D_{f} + M \times N \times D_{f} \times D_{f} .$$

In our study, $D_{k}$ was set to 3. According to Hollemans [34], and having the same number of input and output channels is beneficial for increasing computational speed and reducing memory overhead. Therefore, $M$ and $N$ were both set to 32 for down-sampling and up-sampling. Compared with the standard convolution, the number of parameters for separable convolution dropped from 9216 to 1312.

#### Spatial attention module

Prior research has found that placing the targeted tree in the middle of the image can reduce image distortion and the influence of complex backgrounds on the extraction of the tree trunk, thereby increasing the measurement accuracy [3]. Therefore, we proposed that the device be placed such that the area to be used for the DBH measurement was in the middle of the photo when measuring. This allowed us to use the special features of a captured photo to filter out non-targeted trees and the background, further improving the accuracy of object detection. Based on this, we adopted the spatial attention module proposed by Woo et al. [35] to make the CNNs focus on processing the middle area of the photo, which utilizes both max-pooling and average-pooling operations along the channel axis to process the prior channel-refined feature maps and concatenate them to generate an efficient feature descriptor. On the concatenated feature descriptor, a convolution layer and a sigmoid function were applied to generate a spatial attention map that determines areas to emphasize or suppress.

#### General architecture of CNNs

To meet the requirements for semantic edge detection, U-net utilizes convolution up-sampling to maintain the resolution of the output image, which is consistent with the input image [36]. Furthermore, U-net added “Concatenation” into the computation process, which connects prior feature maps with the semantic features, enabling valid features to be reused by the networks, thereby strengthening the learning ability of the networks.

In our research, we used a similar architecture to U-net, consisting of a contracting path and an expansive path [36]. The contracting path is composed of the repeated application of 3 × 3 convolutions, followed by a rectified linear unit and a 2 × 2 max-pooling operation for down-sampling [36]. The number of feature channels was doubled at each down-sampling step. In the expansive path, each step includes an up-sampling of the feature map, followed by an up-convolution that halves the number of feature channels [36]. A “Concatenation” operation was then used to connect with the corresponding cropped feature map in the contracting path, along with two 3 × 3 convolutions, each followed by a rectified linear unit. Cropping was required to account for the loss of border pixels in each convolution [36]. Between the third convolution pool layer and the first convolution layer, we used a spatial attention module to optimize the network outputs. In the final layer, we used a 1 × 1 convolution to map each feature vector to the desired object class. All the convolution layers were performed using separable convolution filters.

### Hardware components

The key components and workflow of the proposed device are shown in Figs. 1 and 2, respectively. The major hardware includes an embedded development board, a laser rangefinder, digital camera, GPS, memory module, an LCD, and a battery.

#### Embedded development board

We selected Raspberry Pi 3B^+^ as the development board for our device. Raspberry Pi 3B^+^ possesses a 4-core A53 series Advanced RISC Machines chip, which consumes less power and can reduce the need for a larger battery capacity, thus reducing the size and weight of the device. Furthermore, the Raspberry Pi series has a complete application ecosystem that can fulfill the operating environment required for computer vision and deep learning.

#### Laser rangefinder

Considering the required portability of the device and aiming to minimize the ranging error caused by shaking and trembling, it was necessary to choose a laser ranging module with low power consumption, a small size, and a high measurement frequency. Here, we selected the VL53L1X module as the rangefinder. The measurement range of the VL53L1X module was 5–400 cm, and the measurement frequency was 50 Hz. Its power consumption was 20 mW, and the relative measurement error was approximately 3%.

#### Digital camera

To fulfill the requirements for measurement accuracy, we chose Raspberry Pi Camera V2 to record images of tree trunks. The focal length of the lens of the camera was 16 mm. The sensor had 8 million pixels, and the pixel size was 3.7 μm. The digital camera allowed the video stream data to be collected at 1080 progressive scanning (P)/30 frames per second (FPS).

#### GPS

The selected GPS module possesses good compatibility with Raspberry Pi, which is characterized by its small size and low power consumption. The working current for the GPS module was only 20 mA, the sensitivity was − 165 decibel relative to one milliwatt (dBm), and the data update rate was 1 Hz.

#### Memory module

For Raspberry Pi 3B^+^, the operating system was written onto the secure digital memory card (SD Card). We chose a SanDisk memory card with 32 GB of data storage space as the memory module. The data collected with one shot of the device included a JPG image and a text file for each measured tree. The JPG image data was about 20 KB, and the text file was approximately 1 KB. Theoretically, the device can store the measured data of more than one million trees. Two universal serial bus (USB) ports were used to allow connections to an external flash disk. The device identified and connected with an external disk and transferred the data to the disk when the virtual button “ReadData” on the LCD was pressed.

#### LCD

The Raspberry Pi 3B^+^ has a variety of ways to output the video, such as general purpose input/output (GPIO), USB, and high definition multimedia interface (HDMI). Considering the need to shoot the tree in real time, we selected an LCD with an HDMI interface, which has a resolution of 800 × 480 and a refresh rate of 30 FPS.

#### Battery

The capacity of the selected lithium battery was 12,000 mA, and the voltage was 3.7 V. When the device was powered on, the idle current was approximately 850 mA, the working current was approximately 1000 mA, the peak current was approximately 1200 mA, and the theoretical working time was approximately 12 h.

### Graphical user interface (GUI) and operation guide

#### Take–save

**Fig. 4:**
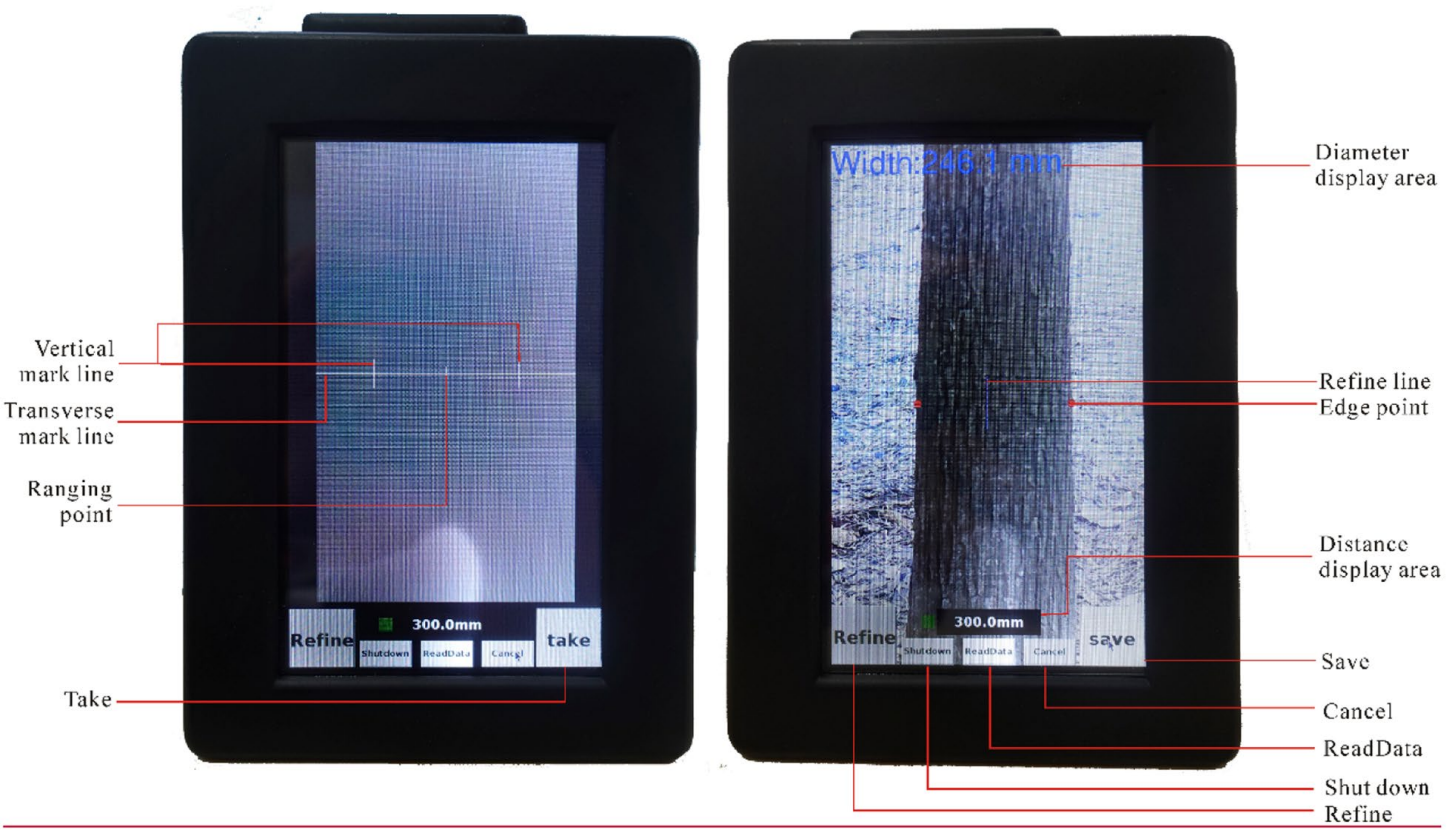
Graphical user interface of the presented device

Pressing the virtual button “Take” (Fig. 4) caused the device to take a photo of the targeted tree trunk, measure the horizontal distance between the camera and the targeted tree trunk, and calculate the DBH. Then, “Take” will change into “Save,” and pressing the “Save” button saves the measured DBH value. The recorded data was written into a text file and stored in the memory card.

#### Vertical mark line

Three vertical lines in the central area of the LCD were used to mark the optimal location of the targeted tree trunk in the recorded image (Fig. 4). According to the measurement accuracy test, the measurement error of DBH is smallest when the proportion of the targeted trunk on the image is 50–60% (Fig. 9). In the GUI design, when the targeted trunk is located between the right and left vertical mark lines, the proportion of the tree trunk on the image is approximately 50–60%. When taking DBH measurements, it is good practice to place the tree trunk between the two vertical mark lines in order to obtain the best detection accuracy.

#### Transverse mark line

A transverse line located in the middle of the LCD was used to mark the position of the DBH measurements (Fig. 4).

#### Ranging point

The ranging point is the crossing point of the transverse and the central vertical mark lines. When taking DBH measurements in practice, the distance used to calculate the DBH value is the measured horizontal distance between the laser rangefinder and the ranging point.

#### Edge point

Two red points show the edges detected by the CNNs at the measuring position (Fig. 4).

#### Refine and refine line

Pressing the “Refine” button will let the device enter into the “Refine mode.” The operator can redefine the trunk edges manually if obvious errors occur in the automated edge detection. In the “Refine mode,” you can press the screen with your finger to move the “Refine line” (two vertical blue lines used to mark and correct the detected trunk edges on the left and right) (Fig. 4). The “Refine line” will move with your finger until the “Refine line” reaches the edge of the tree trunk.

#### Cancel

If you press the “Cancel” button, the newly measured data and the photos taken by the device will not be stored in the memory card (Fig. 4).

#### Diameter display area

This area was used to show the measured value of DBH (Fig. 4).

#### Distance display area

The measured distance between the camera and the targeted tree trunk in this area is shown in Fig. 4.

#### ReadData

When the device is connected to an external USB storage device, pressing “ReadData” will allow the measured data to be transferred to the external USB storage device. A folder named “data” will be created in the external USB storage device, which contains two sub-folders named “record” and “images,” respectively. The measured DBH values will be stored in the “record” folder, and the photos of tree trunk will be stored in the “images” folder. The format of the photographs was JPG. The measured DBH value, longitude, and latitude were written in a text file. Both the JPG file and the text file were named using the imaging time (for example, 2019-12-01-08-17-50.jpg and 2019-12-01-08-17-50.txt, where 2019 is the year, 12 is the month, 01 is the day, 08 is the hour, 17 is the minute, and 50 is the second).

#### Shut down

This button was used to power off the device safely, with the measured data safely stored in the memory card.

### Evaluation of the accuracy of DBH measurement

Two methods were employed to evaluate the measurement accuracy of the proposed device. The first method utilized the known diameter values (50 mm, 100 mm, 150 mm, 200 mm, 250 mm, and 300 mm) of six standard cylinders as a reference. For each standard cylinder, we first measured the diameter ten times at a distance of 2 m away from the standard cylinder, and then repeated the measurement at a distance of 2.5 m and 3 m using our device. Then, to determine the optimal viewing frame, we analyzed the relationship between the absolute relative error (absRE) of DBH measurement and the percentage of the targeted trunk on the acquired image.

The second method used the diameter values measured using conventional diameter tape in the field as a reference to evaluate the measurement accuracy. The measurements were taken in a semi-natural (deciduous broad-leaved) forest park located in the suburban Mentougou district of Beijing. Many trees in this park are natural, but there are also many trees that were planted 20–30 years ago. The test data were gathered during field measurements in December 2019. The measurements were conducted from 14:00 to 16:30, during a cloudless afternoon, with good, but not very strong, sunlight. In total, the DBH values of 121 trees (*Koelreuteria paniculata*, *Ailanthus altissima*, *Robinia pseudoacacia*, and *Fraxinus chinensis*) were recorded using both the newly developed device and the conventional tape.

To evaluate the measurement accuracy, we first calculated the absRE of the diameter values measured by our device and analyzed the distribution of absRE. We also calculated the average absolute relative error (aveRE), BIAS, root mean square error (RMSE), relative BIAS (relBIAS), and relative RMSE (relRMSE) to evaluate the measurement accuracy. BIAS, RMSE, relative BIAS, relative RMSE, absRE, and aveRE are defined by the following equations:

$${\text{absRE}} = \frac{{\left| {x_{i} - x_{ir} } \right|}}{{x_{ir} }} \times 100,$$

$${\text{aveRE}} = \sum\limits_{i = 1}^{n} {{{\left( {\frac{{\left| {x_{i} - x_{ir} } \right|}}{{x_{ir} }} \times 100} \right)} \mathord{\left/ {\vphantom {{\left( {\frac{{\left| {x_{i} - x_{ir} } \right|}}{{x_{ir} }} \times 100} \right)} n}} \right. \kern-\nulldelimiterspace} n}} ,$$

$${\text{BIAS}} = \frac{{\mathop \sum \nolimits_{i = 1}^{n} \left( {x_{i} - x_{ir} } \right)}}{n},$$

$${\text{relBIAS}} = \frac{{\mathop \sum \nolimits_{i = 1}^{n} \left( {\frac{{x_{i} }}{{x_{ir} }} - 1} \right)}}{n} \times 100,$$

$${\text{RMSE}} = \sqrt {\frac{{\mathop \sum \nolimits_{i = 1}^{n} \left( {x_{i} - x_{ir} } \right)^{2} }}{n}} ,$$

$${\text{relRMSE}} = \sqrt {\frac{{\mathop \sum \nolimits_{i = 1}^{n} \left( {\frac{{x_{i} }}{{x_{ir} }} - 1} \right)^{2} }}{n}} \times 100.$$

Here $x_{i}$ is the ith measurement, $x_{ir}$ is the ith reference, and *n* is the number of estimations.

We then calculated the Pearson correlation coefficient between the reference values and the diameter values measured by our device using bivariate correlations (2-tailed), and compared the measured diameter values with the reference using an independent sample t-test to determine whether significant differences existed between them. We also conducted a linear regression analysis to test the relationship between the reference and diameter values measured by our device.

## Results

### Compared with the known diameter values of standard cylinders

**Fig. 5:**
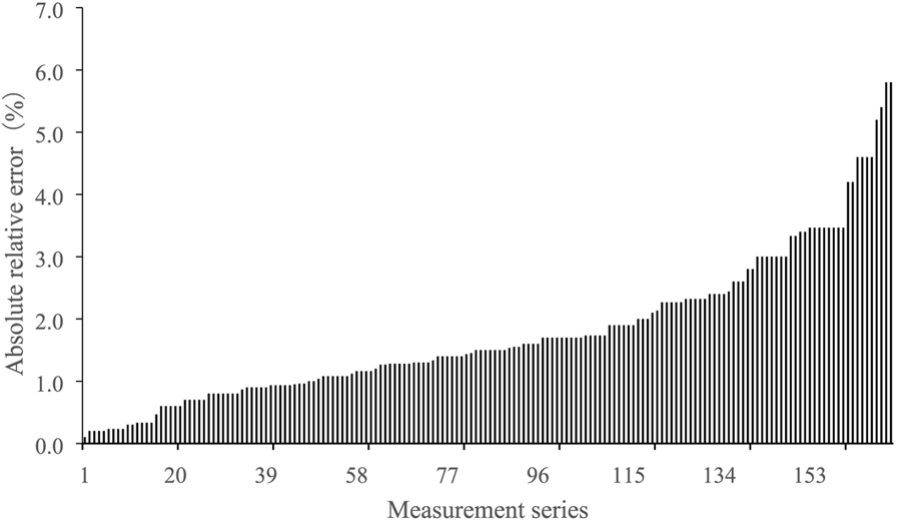
Distribution of absolute relative error (reference is the known diameter values of the standard cylinders)

We found that the absRE ranged from 0.1 to 5.8% (Fig. 5), with an average value of 1.76%, and a median value of 1.5%. The absRE of 87% of the measurements was less than 3%.

**Fig. 6:**
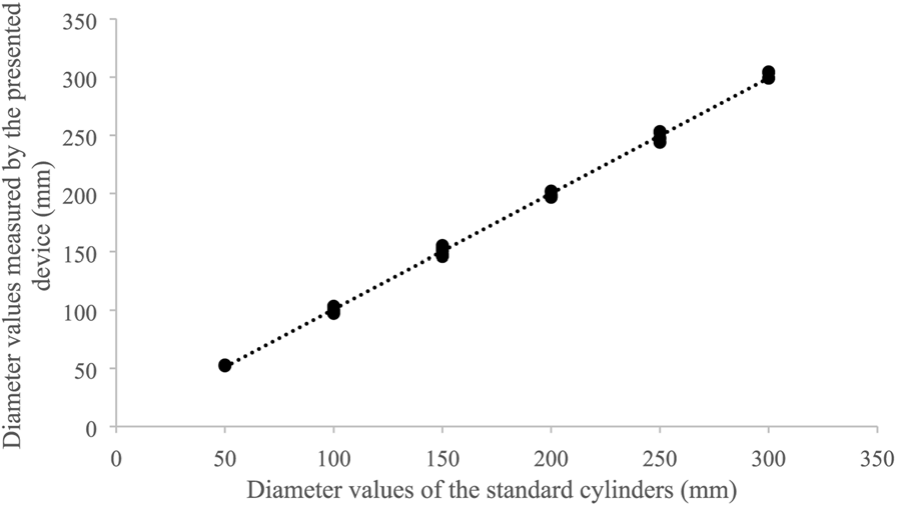
Relationship between the diameter values measured by the presented device and the diameter values of the standard cylinders

Correlation analyse, independent sample t-test, and linear regression indicated that the measured diameter values were significantly correlated with the reference values (Table 1, Fig. 6), and no significant differences existed between the measured diameter values and the reference values (p > 0.01, Table 1).

**Table 1:** Measurement accuracy of the newly developed device

	Diameter values of standard cylinders measured by the device	Diameter values of individual trees measured by the device
aveRE (%)	1.76	3.38
BIAS (mm)	0.064	− 1.78
relBIAS (%)	0.32	− 0.77
RMSE (mm)	3.07	6.36
relRMSE (%)	2.11	4.22
Pearson correlation coefficient	0.999**	0.991**
sig. of independent sample test	0.808	0.772

**Correlation is significant at the 0.01 level (2-tailed). (Diameter values of standard cylinders measured by the device: reference is the known diameter values of standard cylinders; diameter values of individual trees measured by the device: reference diameter being the values measured using conventional tape)

BIAS, relBIAS, RMSE, and relRMSE showed that the diameter values measured by the device were close to the reference values.

### Comparison with the diameter values measured by the conventional tape

**Fig. 7:**
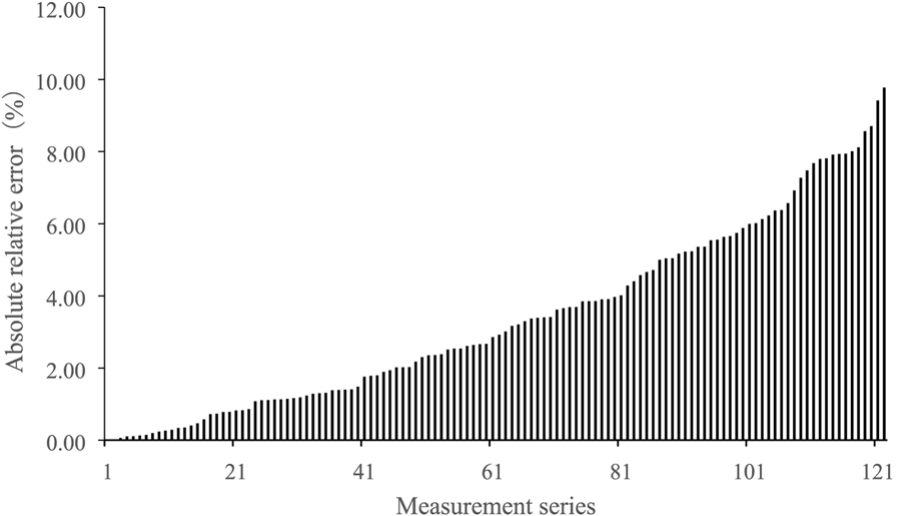
Distribution of absolute relative error (reference diameter being the values measured using conventional tape)

The absRE ranged from 0.0 to 9.77% (Fig. 7), with an average value of 3.38%, and a median value of 2.7%. The absRE of 70% of measurements was less than 5%.

**Fig. 8:**
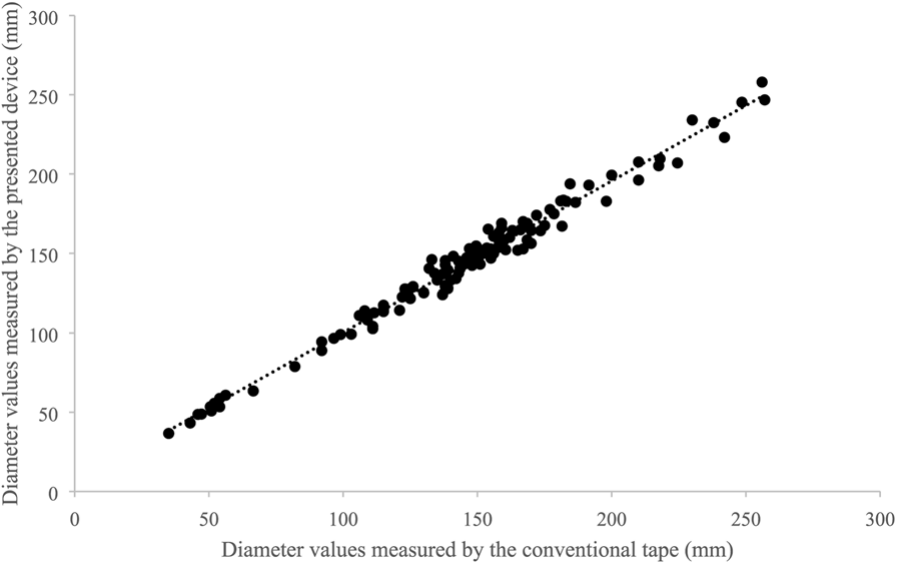
Relationship between the diameter values measured by the presented device and the diameter values measured by the conventional tape

We found that the measured diameter values of the device were significantly correlated with the reference values (p < 0.01, Table 1, Fig. 8), and no significant differences were observed between the diameter values recorded by the device and the reference values (p > 0.01, Table 1).

BIAS, relBIAS, RMSE, and relRMSE showed that the measured diameter values were close to the reference values (Table 1).

### Optimal viewing frame

**Fig. 9:**
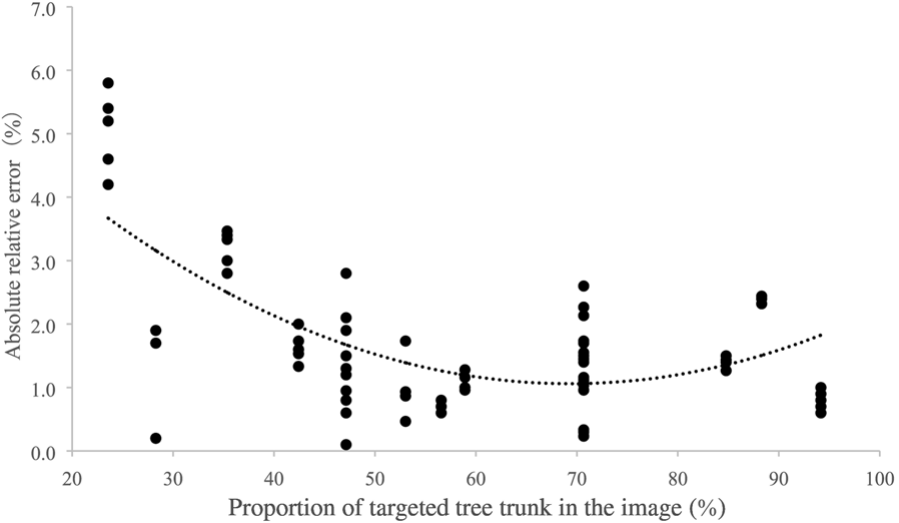
Relationship between absolute relative error and percent of targeted tree trunk in the acquired image

We found that the absolute relative error reached its lowest value when the targeted tree trunk occupied nearly 50–60% of the image (Fig. 9). Based on this, we provided the optimal viewing frame using two virtual vertical lines on the LCD in the graphical user interface design (Fig. 4).

## Discussion

### Characteristics of the presented device

The device developed in our research offers high-level integration to save space, reduce weight, decrease power consumption, and increase battery life, making it a good choice as a battery-powered handheld device for efficient DBH measurement. Moreover, no additional tools, such as reference sticks and height poles are needed in the field measurements of DBH. Most other image-based instruments for DBH measurement are a loose collection of components; auxiliary tools such as tripods and height poles are needed to implement the measuring process [11]. Furthermore, the measured DBH value is presented on the LCD instantly, and no further data processing is needed on the computer. The measured DBH values are also stored in the device in the format of a text file, which can be conveniently transferred to computers or other external storage devices. Our device integrated a GPS receiver that could record latitudes and longitudes of measurement sites. This is critical information for us to understand the spatial distribution pattern of forest resources at a larger geographical scale.

One critical step in DBH measurement is to locate the measuring position on the trunk that is 1.3 m above the ground (the breast height). A bottom-point of the targeted tree is required to determine the breast height and locate the measuring position. Several researchers locate the bottom-point using a manual approach [18], or with the help of a marker [11]. Wu et al. [3] presented a complicated algorithm to determine the measurement position based on the depth information of the tree and spatial coordinate system transformation. However, in practice, it is difficult to guarantee the accuracy of locating the bottom point automatically because of the occlusion of dwarf shrubs, herbs, rocks, and the curve of the land surface, particularly in thick tropical or sub-tropical forests. The uncertainties in bottom-point determination will definitely influence the accuracy of the DBH measurements. Our device does not provide an automatic location for the measuring position.

Many image-based devices can measure not only the DBH, but also the tree height and crown size [3, 11, 18]. Tree height measurement is a difficult task in forest surveys owing to the difficulty in locating the vertices of trees, particularly in dense forests of tropical or sub-tropical regions. In addition, it is difficult to obtain a photo of the entire tree crown in the thick forest. Hence, our device is not a “Jack of all trades,” as it focuses on the DBH measurement.

### Tree trunk detection based on CNNs

In our research, we utilized CNNs to detect the trunk of a targeted tree. We also adopted a spatial attention module to increase the accuracy of object detection in complex environments. We found that in most cases, the object detection algorithm based on CNNs could identify the trunk fairly well for barks with uniform color and consistent surface texture. However, sometimes it caused obvious errors in the detection of tree trunk when the barks were variegated, rough, and shaggy, particularly when there were spots of sunlight on the tree trunk. To deal with this problem, we designed the “Refine” module to correct the error of edge detection manually. Furthermore, measures of image processing should be conducted to decrease the effects of sunlight on object detection. In addition, the amount of training samples also had a critical influence on the performance of deep-learning-based object detection algorithms. In our research, only 100 pictures of five species were used to train the CNNs. This definitely limited the ability of CNNs for object detection. Therefore, in the future, more pictures including more tree species should be collected to enhance the object detection ability of CNNs.

We also planned to increase the number of training samples by building a “Treebank” database. The pictures taken by the device in the field will be transferred to the “Treebank” wirelessly. These pictures will be used for training the model on the “Cloud” (Server), and the trained model (CNNs) will be used for tree measurement. With the increase of picture numbers in the “Treebank,” the detection ability of CNNs will be continuously improved. The limitation for this technique is the availability of internet, but it is fortunate that mobile 4G signal is almost everywhere.

### Evaluation of measurement accuracy

The correlation analysis and independent sample t-test indicated that the DBH values measured by the newly developed device were significantly correlated with the references, and no significant differences (p > 0.01) were detected between the measured DBH values using our device and the references. This verified that the presented device could satisfy the requirements of field forest surveys.

The accuracy of previous image-based DBH estimations varied greatly. Varjo et al. [13] developed an image-based method that resulted in a − 0.6 mm to − 2.8 mm BIAS, and 7.0–9.4 mm RMSE in DBH estimates for trees with different heights. Liang et al. [15] estimated the DBH using images with the G (green) channel and the RGB channels. The BIAS and relBIAS were 4.8 mm and 1.33% for DBH values estimated from the G image, and 19.8 mm and 5.39% for the RGB image, respectively. The RMSE and relRMSE were 23.9 mm and 6.6% for DBH values estimated from the G image, and 44.7 mm and 12.14% for the RGB image, respectively. Adilson et al. [37] utilized vertical fisheye images to measure the DBH and achieved an RMSE of 14.6 mm. In the study by Fan et al. [18], the RMSE of DBH estimations using smartphones was 12.6 mm, relRMSE was 6.39%, BIAS was 3.3 mm, and relBIAS was 1.78%. Wu et al. [3] developed a smartphone-based DBH measuring device, and the RMSE of the measured DBH values was 2.17 mm. Compared with most previous studies, our device showed higher measurement accuracy (Table 1).

In the field measurement for accuracy testing using the newly developed device, we took only one picture for each tree from one direction to estimate the DBH of the targeted tree. This definitely influenced the DBH measurement accuracy because the tree trunk is not a standard cylinder and the cross-section is not a perfect circle. The measured points represent different diameters with any change in view angle [8]. Hence, we proposed to perform repeated measurements from multiple directions to improve the measurement accuracy when using the newly developed device in the field DBH measurements.

Here, we need to emphasize that the measurement accuracy reported in our research was based on data collected in a deciduous broad-leaved forest in winter. If the test measurement was taken in summer or autumn, the measurement accuracy might be slightly different from that in the winter, as the light condition may vary under the forest. In addition, the measurement site is located in a semi-natural forest, where the tree density may be lower than in a natural forest, particularly sub-tropical or tropical forests. This means that the background of the targeted tree at our test site might be less complex than that of a natural forest. Previous research has proved that complex backgrounds have a negative influence on the extraction of the tree trunk [3]. Therefore, the measurement accuracy of the new device may change slightly if the test data are collected in a natural forest, due to the variation in forest structure.

### Economic cost of the newly developed device

Among the dendrometers applied in forest surveys, the cheapest is the conventional tape, which costs less than five dollars for  a tape in China. The LiDAR system is a popular high-throughput technique for DBH measurements. However, the LiDAR system is expensive, costing about fifty thousand dollars to buy the instrument. Another high-throughput technique for DBH measurements is the image-based point-cloud data method. In this method, you need one or several cameras to gain multiple images of the plot from different directions, with the price for one camera ranging from five hundred dollars to six thousand dollars, or even more. For our device, the hardware cost about two thousand dollars. If the device were mass produced, the cost would decrease to five hundred dollars per device.

Although the economic cost of the new device is lower than that of the LiDAR system, it does not mean that the new device could replace the LiDAR system. This is because the design and application scenario of the new device are different from those of LiDAR. Our device is suitable for diameter measurements of individual trees at the quadrat level (usually 20 m × 30 m). Whereas the LiDAR is usually used to measure the diameters of trees at plot level (100 m × 100 m, or even larger).

### Analysis of major error sources

The horizontal distance between the laser rangefinder and the targeted tree is a very important factor that affects the measurement accuracy. However, the coded spot emitted by a laser is easily flooded by sunlight [3]. When the device faces the sun or specular light, the ranging precision cannot be guaranteed.

The newly developed device is a handheld instrument, and no tripod and spirit level are required for measurement. It is convenient and efficient to conduct measurements in the field. However, without the support of a tripod and spirit, it is difficult to keep the device parallel to the targeted tree trunk. This may lead to an error in distance measurement and affect its accuracy.

Errors also occurred in the detection of trunk edges, which cause further errors in determining the pixel number between the edges of the tree trunk, leading to the detriment of the measurement accuracy.

The trunk form also causes a difference between the DBH values measured by the new device and that of the conventional tape. We have previously discussed this issue in “Discussion”, and more details were presented in “Evaluation of measurement accuracy” section.

## Proposed application scenarios

Our device is designed to provide an alternative to conventional tape, or to replace it in the measurement of tree diameter. It is suitable for measuring the DBH of individual trees in forest inventory at the quadrat level. Our device can also be applied in forestry and agriculture related industries, such as for the measurement of plant traits in plant breeding, as it can be an efficient measurement of tree diameter and fruit diameter.

## Conclusion

The newly developed handheld device realized efficient, accurate, instant, and non-contact measurements of DBH, and the CNNs were proven to be successful in the detection of tree trunks in our research. The measured diameter values and the recorded longitudes and latitudes of the measurement sites were written into a text file, which was convenient for export to an external flash disk. We believe that the newly developed device can fulfill the precision requirement in forest surveys, and that the application of this device can improve the efficiency of DBH measurements in forest surveys.

## Data Availability

The data are available upon reasonable request to the authors.

## Abbreviations

DBH: Diameter at breast height
3D: 3-Dimension
CNNs: Convolutional neural networks
GPS: Global positioning system
LCD: Liquid crystal display
USB: Universal serial bus
ARM: Advanced RISC Machines
SD Card: Secure digital memory card
GB: Giga Byte
KB: Kilo Byte
JPG: Joint Photographic Experts Group
CCD: Charge Coupled Device
mW: Milli Watt
Hz: Hertz
FPS: Frame Per Second
mA: Milli Ampere
dBm: Decibel relative to one milliwatt
HDMI: High definition multimedia interface
GPIO: General purpose input/output
GUI: Graphical user interface
absRE: Absolute Relative Errors
aveRE: Average absolute Relative Error
RMSE: Root Mean Square Error
relBIAS: Relative BIAS
relRMSE: Relative RMSE
RGB: Red Green Blue
